# Association between changes in cerebral grey matter volume and postoperative cognitive dysfunction in elderly patients: study protocol for a prospective observational cohort study

**DOI:** 10.1186/s12871-016-0285-z

**Published:** 2016-11-25

**Authors:** Nicolai Goettel, Panagiota Mistridis, Manfred Berres, Julia Reinhardt, Christoph Stippich, Andreas U. Monsch, Luzius A. Steiner

**Affiliations:** 1 Department of Anesthesia, Surgical Intensive Care, Prehospital Emergency Medicine and Pain Therapy, University Hospital Basel, University of Basel, Spitalstrasse 21, CH-4031 Basel, Switzerland; 2 Department of Clinical Research, University Hospital Basel, University of Basel, Basel, Switzerland; 3 Memory Clinic, University Center for Medicine of Aging Basel, Felix Platter Hospital, Basel, Switzerland; 4 Department of Mathematics and Technology, University of Applied Sciences Koblenz, Koblenz, Germany; 5 Department of Radiology, Division of Diagnostic and Interventional Neuroradiology, University Hospital Basel, University of Basel, Basel, Switzerland

**Keywords:** Postoperative cognitive dysfunction, Long-term outcomes, Surgery, Anesthesia, Cerebral volume

## Abstract

**Background:**

Cognitive decline is frequently observed in elderly patients after major surgery. The pathophysiology of postoperative cognitive dysfunction (POCD) remains unclear. The aim of our investigation is to identify potential associations between brain volume change and POCD in elderly patients undergoing major surgery.

**Methods:**

This is a prospective observational cohort study approved by the regional ethics board. We intend to compare specific brain volumes (hippocampus, lateral ventricle, total grey matter volume, regional cortical thickness) on magnetic resonance imaging and cognitive functions determined by a neuropsychological assessment battery in 70 study participants aged ≥65 years before and 3 and 12 months after major noncardiac surgery. Thirty volunteers will be included as matched nonsurgical controls. The primary endpoint of the study is the change in hippocampal volume over time in patients with and without POCD. The secondary endpoint is the correlation between the change in cerebral volume and cognitive function. We will follow the STROBE guidelines for reporting the results of observational studies.

**Discussion:**

We hypothesize that surgery under general anesthesia is associated with a loss of cerebral grey matter, and that the degree of postoperative cognitive dysfunction correlates with the extent of atrophy in areas of the brain that are relevant for cognitive functions. The validation of reproducible anatomical biomarkers, such as the specific brain volumes examined in our cohort, may serve to evaluate the effect of preventive strategies and treatment interventions for POCD in follow-up studies.

**Trial registration:**

Clinicaltrials.gov NCT02045004. Registered 22 January 2014. Kofam.ch SNCTP000001751. Registered 21 April 2016 (retrospectively registered).

## Background

Anesthesia care for patients in their seventies or eighties has an ever-growing presence in clinical practice given the demographic development in many industrialized countries. Variable degrees of cognitive decline are frequently observed after major surgery in elderly patients and have been defined as postoperative cognitive dysfunction (POCD) [1]. Clinical studies have reported an incidence of POCD of up to 41% in surgical patients older than 60 years (Table 1) [2]. In many patients, this is a transient problem. But in some patients, cognitive deficits are long-term or permanent [1, 2]. POCD may also develop in younger patients [3] and after minor surgery [4]. The occurrence of POCD is associated with increased mortality and important socioeconomic consequences [5].

**Table 1:** Incidence of POCD after noncardiac surgery

Study	Population	Time for postoperative test	POCD rate (%)
Moller et al. [1]	Patients ≥60 undergoing major abdominal, thoracic, or orthopedic surgery	1 week and 3 months	25.8 and 9.9%, respectively
Monk et al. [2]	Patients >60 undergoing minimally invasive, intraabdominal/thoracic, or orthopedic surgery	1 week and 3 months	41.4 and 12.7%, respectively
Johnson et al. [3]	Patients 40–60 undergoing major abdominal or orthopedic surgery	1 week and 3 months	19.2 and 6.2%, respectively
Canet et al. [4]	Patients >60 undergoing minor surgery	1 week and 3 months	6.8 and 6.6%, respectively

*POCD* postoperative cognitive dysfunction

The potential association between exposure to anesthesia and the development of dementia in elderly patients remains a controversial topic [6, 7]. So far, a direct association between surgery and long-term cognitive decline has not been established [8]. The pathophysiology of POCD is poorly understood, and no preventive strategy or treatment has been described so far [9]. A retrospective analysis of cohort data suggests that surgery may be associated with a decrease in brain volume [10]; however, no analysis of a potential link between cerebral atrophy and cognitive functions was made. We would like to reproduce and verify these results and investigate a possible relationship with the postoperative cognitive performance in surgical patients.

The principal objective of this observational cohort study is to prospectively test the hypothesis whether major noncardiac surgery under sevoflurane-based general anesthesia is associated with a loss of cerebral grey matter in elderly patients. We also hypothesize that the degree of POCD correlates with the loss of grey matter in brain areas relevant for cognitive functions.

## Methods

### Study design and setting

This prospective, observational cohort study is being conducted at the University Hospital Basel, Switzerland. Study recruitment commenced in July 2015 and is expected to last for 36 months. The regional ethics committee (EC) approved the study prior to participant inclusion (Ethikkommission Nordwestschweiz, protocol: EKNZ 2014–155, 1 August 2014). The study was registered on clinicaltrials.gov (NCT02045004) on 22 January 2014 and on Kofam.ch (SNCTP000001751) on 21 April 2016.

### Study population

Two groups of study participants will be investigated in this prospective cohort study. Seventy patients aged ≥65 years undergoing major surgical procedures will be recruited for this study (Group 1). Thirty volunteers aged ≥65 years will be recruited as matched nonsurgical controls (Group 2).

#### Recruitment and informed consent

Study participants in the surgical group (Group 1) will be recruited as patients of the University Hospital Basel, Switzerland. Eligible patients are identified by screening the daily list of visits in the preoperative anesthesia clinic. They are either contacted personally on the same day, or by mail in a letter including participant information and a consent form. A follow-up phone call by an investigator will provide further information about relevant details of the study. Study participants in the nonsurgical control group (Group 2) will be recruited from an existing study subject registry established by the Memory Clinic at the Felix Platter Hospital, Basel, Switzerland. Nonsurgical controls are matched to surgical patients according to age, gender and level of education.

The study is conducted with written informed consent from all study subjects and is conducted with respect of the most recent version of the Declaration of Helsinki [11]. Participants are informed that participation in the study is voluntary, and that they are free to withdraw at any time. Recruitment and consenting of study participants by members of the research team is in-line with Good Clinical Practice (GCP) [12].

#### Inclusion and exclusion criteria

Study inclusion and exclusion criteria appear in Table 2. All patients aged 65 years or older, American Society of Anesthesiologists’ physical status I–III, who are scheduled from major elective surgery under general anesthesia at the University Hospital Basel, are eligible for study inclusion in Group 1. Further inclusion criteria comprise a home location suitable for testing, German as their first language, and an Instrumental Activities of Daily Living [13] score ≥6. Participants in Group 2 are volunteers who present equal inclusion and exclusion criteria, without planned surgery.

**Table 2:** Study inclusion and exclusion criteria

Criterion	Screening procedure
Inclusion criteria	
Age ≥65 years	Medical record
Home location suitable for testing	Medical record
German as a first language	Medical record or recruitment telephone call
IADL score ≥6	Baseline assessment
ASA physical status I–III	Baseline assessment
Major elective surgery^a^	Medical record
Planned general anesthesia^a^	Baseline assessment
Exclusion criteria	
Cardiac surgery^a^	Medical record
Neurosurgery including carotid endarterectomy, or any type of surgery precluding postoperative testing^a^	Medical record
(Another) major surgery within the study timeline	Medical record, recruitment telephone call or baseline interview
General anesthesia up to 3 months prior to inclusion	Medical record
Baseline MMSE score <24	Baseline neuropsychological assessment
Dementia criteria per DSM-5	Baseline neuropsychological assessment
Previous pathological cerebral imaging (if available)	Medical record
History of cerebral or cerebrovascular pathology, head trauma, neurodegenerative illness or epilepsy	Medical record
Chronic use of psychiatric medication	Medical record or baseline interview
Alcohol or substance abuse	Medical record or baseline interview
History of chronic pain unrelated to the planned surgery	Medical record
Chronic medical illness known to induce encephalopathy	Medical record
Any contraindication for MRI (e.g., pacemakers and other MRI-incompatible implantable device)	Medical record, recruitment telephone call or baseline interview
Claustrophobia	Medical record, recruitment telephone call or baseline interview
Incidentally diagnosed disease or unfavorable course of disease in participants who choose not to be informed	Any study assessment
Lack of informed consent	Recruitment telephone call

*ASA* American Society of Anesthesiologists, *DSM-5* Diagnostic and Statistical Manual of Mental Disorders, Fifth Edition, *IADL* Instrumental Activities of Daily Living, *MMSE* Mini-Mental State Examination

^a^Not applicable for the nonsurgical control group

### Outcomes

The primary endpoint of this study is the degree of change in hippocampal volume over time between patients with and patients without POCD. Other specific cerebral volumes measured are the lateral ventricles, the total grey matter volume, and regional cortical thickness. The secondary endpoint is the correlation between the change in cerebral volume and the change in cognitive function.

### Study procedures

**Fig. 1:**
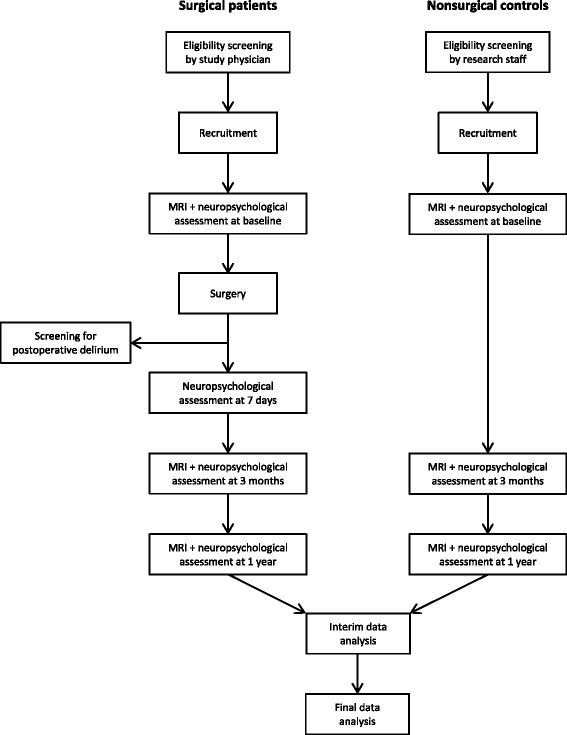
Flow diagram of study procedures

Study-related procedures include a baseline multimodal assessment, intraoperative data acquisition and postoperative multimodal assessments on study visits scheduled 1 week, 3 months and 1 year after surgery. Figure 1 shows the flow diagram for the study assessments on a time line.

#### Anesthetic management

Anesthesia will be standardized. Intravenous propofol and fentanyl will be used for induction of general anesthesia, atracurium or rocuronium for neuromuscular blockade. Patients will be intubated and mechanically ventilated with an air/oxygen mixture to maintain an end-tidal carbon dioxide at 4.6 ± 0.6 kPa. Inhalational sevoflurane, intravenous fentanyl or remifentanil will be used to maintain anesthesia. Intraoperatively, the dose of anesthetics will be controlled using depth of anesthesia monitoring at a targeted bispectral index (BIS) of 40–60 and end-tidal sevoflurane concentration monitoring. Data will be downloaded directly from the patient monitor (IntelliVue™ MX800, Philips Healthcare, Zurich, Switzerland) and the anesthesia machine (Perseus™, Dräger, Liebefeld, Switzerland) on a personal computer using ICM+ software (Cambridge Enterprise Ltd, University of Cambridge, United Kingdom).

#### Neuropsychological assessment

The Consortium to Establish a Registry for Alzheimer’s Disease-Neuropsychological Assessment Battery (CERAD-NAB) [14], Trail Making Tests A and B [15], and phonemic fluency (s-words) [16] will be performed preoperatively, at 7 days, 3 months and 1 year postoperatively. Training of all study personnel and supervision of cognitive testing will be carried out by the Memory Clinic at Felix Platter Hospital. Cognitive functions will be quantified using the CERAD-NAB total score [17], in its demographically-corrected form [18]. A correction for short-term practice effects will be carried out based on previous work [19]. Table 3 shows the neuropsychological subtests used in this study [20]. The 15 variables resulting from neuropsychological assessment are converted into standard scores (z-scores) based on a normative sample [21], and the z-score changes from baseline are calculated. POCD is diagnosed if the decline is >1.0 standard scores in two or more of the 15 variables based on the diagnostic guideline for mild cognitive impairment and dementia related to Alzheimer’s disease by the National Institute on Aging-Alzheimer’s Association [22] and the American Psychiatric Association’s Diagnostic and Statistical Manual of Mental Disorders (DSM-5) [23]. Postoperative delirium is assessed using the Delirium Observation Screening scale [24] and, if applicable, the Confusion Assessment Method [25], administered 3 times daily until the fifth postoperative day.

**Table 3:** Subtests of the neuropsychological assessment battery used for diagnosis of POCD

Test variable	Test description	Domain tested
CERAD-NAB word list-encoding	Total number of correctly learned words across three learning trials (number of words per trial = 10)	Verbal episodic learning
CERAD-NAB word list-delayed free recall	Total number of correctly remembered words after a delay following encoding	Verbal episodic memory
CERAD-NAB word list-savings	Proportion of correctly recalled words during delayed free recall relative to words learned in learning trial 3	Verbal episodic memory
CERAD-NAB word list-discriminability	Rate of correctly recognized words from encoding	Verbal episodic memory
CERAD-NAB word list-intrusion errors	Total number of intrusions committed during word list-encoding and -delayed free recall	Executive functions
CERAD-NAB figures-copy	Copy of four figures (circle, diamond, overlapping rectangles, cube)	Visuospatial ability
CERAD-NAB figures-delayed recall	Number of correctly reproduced figures from figures-copy following a delay	Visual memory
CERAD-NAB figures-savings	Proportion correctly reproduced figures at figures-delayed recall relative to figures-copy	Visual memory
Semantic fluency-animals	Number of animals produced within 1 min	Semantic memory
Boston naming test (15-items)	Number of spontaneously correctly named black and white line drawings (maximum = 15)	Language
Trail making test A	Time required to connect circles numbered from 1 to 25 in ascending order	Psychomotor speed
Trail making test B	Time required to connect circles containing numbers (1–13) and letters (A-L) in ascending and alternating order	Executive functions
Phonemic fluency	Number of words starting with the letter S produced within 1 min	Executive functions

*CERAD-NAB* Consortium to Establish a Registry for Alzheimer’s Disease-Neuropsychological Assessment Battery. Adapted from Mistridis et al. [20]

#### Other assessments

Other assessment tools include the Charlson Comorbidity Index [26]; the Instrumental Activities of Daily Living [13], the Geriatric Depression Scale [27], and the Short Form Health Survey [28], and will be assessed at baseline, at 3 months, and 1 year postoperatively. Subjective grading of cognitive functions and subjective grading of pain are determined on numeric rating scales at baseline, 7 days, 3 months and 1 year postoperatively. Subjective grading of change in cognitive functions (five-point Likert scale) and subjective grading of successful surgery (yes/no) are assessed at 7 days, 3 months and 1 year postoperatively.

#### Magnetic resonance imaging

Cerebral MRI will be performed at baseline, at 3 months, and 1 year postoperatively on the same day as the neuropsychological assessment. MRI analysis will be carried out by the Division of Neuroradiology at the University Hospital Basel. High-resolution anatomic and diffusion cerebral MRI will be performed using the hospital’s 3-Tesla MAGNETOM Prisma™ MRI scanner (Siemens, Zurich, Switzerland). For MRI analysis, we will assume that the same regions as those described in mild cognitive impairment and Alzheimer’s disease [29] are relevant to POCD. We will perform a region of interest (ROI) analysis (hippocampus, lateral ventricle, total grey matter volume, regional cortical thickness). Cortical reconstruction and volumetric segmentation will be performed with the FreeSurfer software suite [30], which is freely available for download online (http://surfer.nmr.mgh.harvard.edu).

### Statistics

#### Sample size justification

Sample size is estimated based on data describing hippocampal volume. The clinically relevant difference is defined as the difference in hippocampal volume between normal subjects (2.8 ± 0.5 cm^3^) and patients with mild cognitive impairment (2.4 ± 0.4 cm^3^) [29]. Assuming an incidence of POCD of 41% one week after surgery in this age group [2], and a standard deviation of the hippocampal volume change of 0.45 in both patients with and without POCD, a total sample size of 56 subjects may detect a difference of 0.4 cm^3^ in hippocampal volume [29] with a power of 90% at a two-sided significance level of 5%. To compensate for the high loss to follow-up, which is unfortunately typical for studies on POCD [31] and estimated at 20%, we will recruit 70 patients in the surgical group (Group 1). The number of study participants in the nonsurgical control group (Group 2) is arbitrarily set at 30.

#### Statistical analysis plan

The quantitative imaging results will be correlated to the CERAD-NAB total score performance obtained on the same day as the MRI scans. The relationship of changes in volume with intraoperative depth of anesthesia (BIS) and the administered dose of sevoflurane expressed as age-corrected minimum alveolar concentration (MAC) equivalent [32] multiplied by time of administration will be calculated. We will use voxel-wise, multivariate analysis of variance (ANOVA) and the classification/prediction procedure on multi-parameter MRI data in order to build optimal composite predictors of patients’ neuropsychological outcomes. All statistical analyses will be performed using SPSS Statistics software, version 22 (IBM, Inc., Zurich, Switzerland). All graphs will be plotted using Prism, version 6 (GraphPad Software, Inc., La Jolla, CA, USA). An interim analysis will be performed to assess quality of data after primary data acquisition from 20 participants. We will follow the Strengthening the Reporting of Observational studies in Epidemiology (STROBE) statement [33] for all future reports related to this study.

### Data monitoring and stopping rules

The EC agreed that monitoring would be performed by the interdisciplinary study team. This team will evaluate the progress of the trial, verify the accuracy and completeness of the case report forms, and ensure that all protocol requirements and investigator’s obligations are being fulfilled. The progress of the trial is evaluated every 6 months. The investigators may terminate the study prematurely according to certain circumstances (e.g., ethical concerns, insufficient participant recruitment, when the safety of the participants is doubtful or at risk, or when alterations in accepted clinical practice make the continuation of a clinical trial unwise).

### Study safety and reporting of adverse events

There are no specific safety concerns related to this study. During initial screening of participants in the recruitment phase, any subject that presents potential safety concerns related to MRI will be excluded from the study. Above all, MRI does not use ionizing radiation, and there are no known harmful side-effects associated with temporary exposure to the strong magnetic field used by MRI scanners [34]. However, if a serious adverse event should occur, it will be reported to the EC within the appropriate time frame.

## Discussion

This observational cohort study prospectively evaluates the relationship of cerebral grey matter volume and POCD in elderly patients after major noncardiac surgery under sevoflurane anesthesia. The primary hypothesis of this investigation is that POCD is accompanied by a loss of cerebral grey matter. Our secondary hypothesis is that the degree of POCD correlates with the loss of grey matter in brain areas relevant for cognitive functions. There are several potential implications of this study for future clinical practice. The validation of reproducible anatomical parameters or biomarkers, such as the specific brain volumes examined in our cohort, will help to identify high-risk patients and may serve to evaluate the effect of preventive strategies and treatment interventions for POCD in clinical follow-up studies. In summary, findings of this study may contribute to improve the diagnosis and prevention of POCD in the future. In the following, we discuss considerations regarding study design, endpoints, choice of study collective, surgical procedures and anesthetic technique.

### Study design

The cohort study design chosen for our study enables investigative research in a real-life clinical context without influencing state-of-the-art medical care of patients. No study-related intervention is planned in participating patients, and risks are minimal. Given the lack of clinical data and missing preventive and/or clinical treatment approaches towards POCD, our study is aimed to investigate an association between the cognitive decline and an anatomical biomarker. At this point, POCD research is limited to noninterventional clinical studies, hence the prospective observational design of this study.

### Endpoints

The choice of radiological endpoints of our investigation is based on the findings of AddNeuroMed study [29]. Liu et al. reported that the degree of atrophy in specific cortical areas correlates with the results of neuropsychological assessment tests obtained by subjects with various cognitive disorders (unrelated to surgery) [29]. We assume that brain areas affected in POCD are comparable to those concerned in Alzheimer’s disease or mild cognitive impairment.

### Methodological considerations

#### Choice of study collective

Although POCD was described in middle-aged patients [3], the disorder has its highest incidence among patients over the age of 65 years. As most research on POCD was done in this age group, we decided to set the cut-off for participant age at 65 years. We excluded subjects with conditions known to induce changes in cerebral anatomy such as Alzheimer’s disease and other forms of dementia, a history of severe neurological disease, or chronic pain in order to obtain a patient sample free of pre-existing cognitive disorders that might confound the relationship between brain volumes and POCD. The addition of a group of nonsurgical participants serves to control for practice effects related to neuropsychological assessment, to observe the physiological cerebral atrophy rates in volunteers matched to the surgical group, and to establish a paradigm for further research on POCD.

#### Choice of surgical procedures

We carefully considered the range of surgical procedures to include in our study. We excluded cardiac surgery because of potential issues with cardiopulmonary bypass, surgery of the brain or extracranial blood vessels, and any type of surgery of the face or neck that would preclude patients’ active participation in the postoperative neuropsychological assessments. Emergency procedures were also excluded, since we would be unable to measure the patient’s baseline. At our institution, abdominal, gynecologic, urologic, vascular and orthopedic procedures are the most commonly performed major noncardiac surgeries in older adults. Most of these surgeries involve a hospital stay of at least 5 days. Although this study is monocentric, we strongly believe that the resulting study cohort is representative of a general surgical population.

#### Choice of anesthetic technique

In this study with pilot character, we chose to investigate exclusively Sevoflurane-based general anesthesia, in order to standardize anesthetic management as a measure to minimize bias. Sevoflurane anesthesia is delivered by the attending anesthetist primarily based on BIS targets (40–60) and clinical judgement. Given that the choice of anesthetic technique only marginally influences the risk of developing POCD in surgical patients [35], we intend to examine the effect of other anesthetic drugs (e.g., propofol as total intravenous anesthesia) and other anesthetic techniques (e.g., neuraxial or regional anesthesia) on brain volumes and POCD in follow-up studies with similar research protocols. Airway management is standardized as all patients participating in this study are intubated and mechanically ventilated. Other aspects of intraoperative anesthetic management such as the use of opioids or muscle relaxants and hemodynamic management are left at the discretion of the attending physician following existing institutional guidelines.

#### Diagnosis of POCD

POCD is a complex neuropsychological disorder presenting as a decline in various cognitive domains after surgery and may at times be difficult to diagnose. Clinical cognitive outcomes research is complicated by the fact that there is no universal definition of POCD. Assessment for POCD comprises a time-intensive combination of neurocognitive function tests, and expert evaluation is mandatory for a valid diagnosis of POCD. In the literature, large differences in methodology (e.g., the test batteries used, the interval between sessions, the endpoints to be analyzed, statistical methods) and diagnostic criteria for POCD are apparent [31]. In our study, the diagnosis of POCD is based on a decrease of standard scores in the CERAD-NAB.

## Abbreviations

ANOVA: Analysis of variance
BIS: Bispectral index
CERAD-NAB: Consortium to Establish a Registry for Alzheimer’s Disease-Neuropsychological Assessment Battery
DSM-5: Diagnostic and Statistical Manual of Mental Disorders, Fifth Edition
EC: Ethics committee
GCP: Good Clinical Practice
MAC: Minimum alveolar concentration
MMSE: Mini-Mental State Examination
MRI: Magnetic resonance imaging
POCD: Postoperative cognitive dysfunction
ROI: Region of interest
STROBE: Strengthening the Reporting of Observational studies in Epidemiology
